# An Unusual Presentation of Congenital Lobar Emphysema

**DOI:** 10.1155/2017/6719617

**Published:** 2017-04-24

**Authors:** Daniel Arnaud, Joseph Varon, Salim Surani

**Affiliations:** ^1^Dorrington Medical Associates and Universidad Autonoma de Tamaulipas, Houston, TX, USA; ^2^The University of Texas Health Science Center at Houston, Houston, TX 77030, USA; ^3^Texas A&M University, Corpus Christi, TX, USA

## Abstract

Congenital lobar emphysema is an uncommon bronchopulmonary malformation characterized by lobar overinflation and accompanying alveolar septum damage that leads to compression atelectasis of the lung parenchyma and displacement of mediastinal structures, with the resultant ventilation-perfusion mismatch. We present a case of a 33-year-old lady with progressive exertional dyspnea. Chest radiograph findings lead to the suspicion of congenital lobar emphysema, which was then confirmed by a computed tomography (CT) scan. This condition is most commonly identified in newborns, with very few cases being reported in adults. Lobectomy remains the treatment of choice and in general has good outcome.

## 1. Case Presentation

A 33-year-old lady presented to the emergency department with a chief complaint of exertional dyspnea while performing her activities of daily living. This symptom had progressively worsened over a period of two years, to the point of the development of dyspnea at rest. Her past medical and surgical history was unremarkable. She denied any history of smoking or illegal drug abuse; however, she did have exposure to secondhand smoking during her childhood.

On initial physical examination, the patient was comfortable at rest but had significant dyspnea on minimal exertion. The patient's blood pressure was 95/56 mm Hg; heart rate was 83/min; respiratory rate was 18/min (at rest); and oxygen saturation while breathing supplemental oxygen at 28% was 94%. She had decreased chest movement of the right hemithorax with a significant decrease of air entry in that same side; left lung auscultation revealed clear breath sounds.

**Figure 1:**
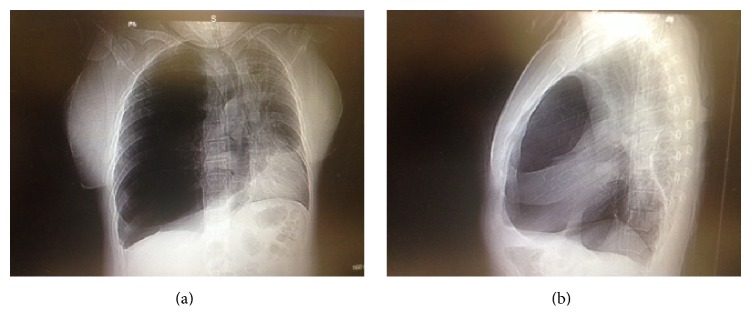
Posteroanterior (PA) chest radiograph depicts an increased translucency of the whole right hemithorax and also severe lateral displacement of the mediastinal structures (a). In addition, the lateral view shows the hyperinflated lobe displacing the oblique fissure and also displacing the mediastinum (b).

**Figure 2:**
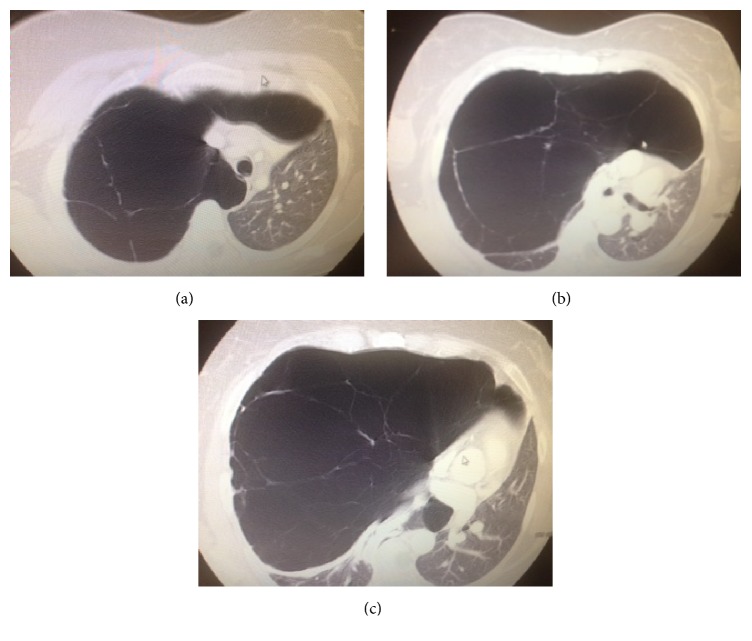
Chest CT scan depicts severe bullous changes, occupying approximately 85% of the right lung and displacing the mediastinum and left lung laterally. Smaller bullous changes are noted in the remaining aerated portion of the right lung. Some portions are significantly compressed between the bullous changes and the aerated portion of the lung. The left lung is also significantly compressed but remains aerated.

A chest radiograph revealed an increased translucency of the whole right hemithorax and lateral displacement of mediastinal structures (see Figure 1). A computed tomography (CT) scan of the chest with contrast was then requested to address the abnormalities noted on chest radiography and depicted severe bullous changes occupying approximately 85% of the right lung and displacing the mediastinum and left lung laterally (see Figure 2). In addition, smaller bullous changes were noted in the remaining aerated portion of the right lung. The left lung was also significantly compressed but remained aerated.

Based on clinical and radiographical findings a diagnosis of right upper lobe congenital emphysema was made. The patient was tested for *α*1-antitrypsin deficiency, which was normal with the phenotype being M_2_ M_2_.

**Figure 3:**
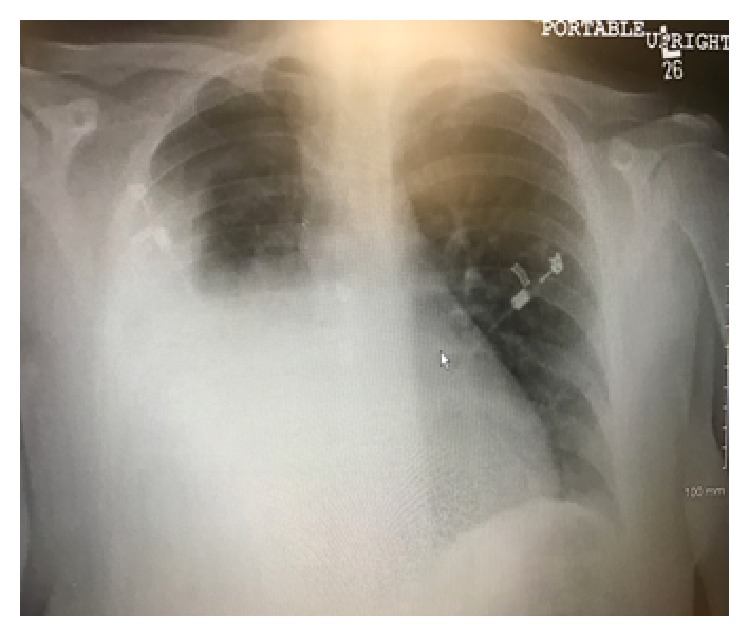
PA chest radiograph showing aeration of right upper lobe and postoperative opacification of the right lower lung field.

**Figure 4:**
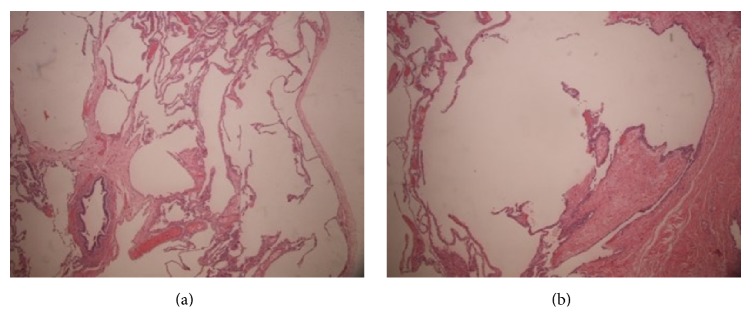
Low and high power view show alveolar space with thin septa and changes consistent with emphysema.

Pulmonary function tests revealed forced vital capacity (FVC) of 1.26 L (37% of predicted) and forced expiratory volume in 1 second (FEV1) of 0.89 L (33% of predicted), with a FEV1/FVC ratio of 70%. Patient total lung capacity (TLC) was 6.2 L (131% of predicted) and a residual volume of 4.93 L (366% of predicted) with corrected diffusion capacity of 75% of predicted. These findings were consistent with severe obstructive lung disease. A quantitative perfusion scan demonstrated 10% contribution from the right lung with 5% from the right upper lobe and 5% from the right lower lobe. The remaining 90% came from the left lung. A 2D echocardiogram revealed no evidence of shunt and an ejection fraction of 55%. The patient was taken to the operating room for an elective right upper lobectomy; however, intraoperatively, the right upper lobe was found to be hypoplastic, and the majority of bullae were coming from the right lower lobe. A right lower lobectomy was performed and postoperatively the patient was rapidly extubated. Postoperative X-ray chest is shown in Figure 3. Pathology specimen showed large dilated alveolar spaces with some containing free-floating septa, which was consistent with emphysematous changes. There was no associated inflammation or malignancy (Figures 4(a) and 4(b)). She had significant improvement in her dyspnea and symptoms. Patient was discharged home on the 5th postoperative day.

## 2. Discussion

Congenital lobar emphysema (CLE) is a bronchopulmonary malformation of unknown cause, with an estimated prevalence of 4.5/100,000 [1]. In general, the male gender is most commonly affected with a ratio of 3 : 1 [2]. In CLE, lobar overinflation is commonly seen, with accompanying damage to the septa that eventually leads to compression atelectasis of the contiguous lung parenchyma, which can result in a shifting of the mediastinal structures, with subsequent ventilation-perfusion inequality [2, 3].

CLE is most frequently identified in full term infants, usually up to 6 months [3, 4]. In adults, this condition is much more uncommon [3, 4]. This clinicoradiological condition is almost always unilateral, and the most frequent affected lobe is the left upper lobe (in up to 43% of cases), followed by the right middle lobe (32%) and right upper lobe (20%) [3, 4]. Lower lobe involvement is very uncommon, as in our case. Multilobar presentation has been reported, but it is very rare [5, 6].

Congenital heart disease has been reported in patients with CLE in about 12–20% of all cases [4, 7]. The most significant and common anomaly found is a left-to-right shunt [3]. For this reason, 2D echocardiogram is suggested, as a part of the initial evaluation of patients with CLE [2, 4].

As noted above, the pathophysiological development of congenital lobar emphysema has not yet ben elucidated, but abnormalities including bronchial cartilage, intrinsic and extrinsic bronchial obstructions, vascular anomalies, alveolar disease, and cytomegalovirus infection have been considered possible factors that can lead to a ball-valve obstruction where a bigger air volume enters the lobe during inhalation than leaves during expiration with following air trapping [1, 8, 9].

The most commonly identified factor in 25% of the patients is congenital bronchial cartilage defects like hypoplasia, flaccid tissue, or absence of cartilage [3]. The remaining percentage has multiple causes of intrinsic or extrinsic obstruction, such as mucus plugging, vascular anomalies, mucosal folds, and/or intrathoracic masses [3].

Emerging data suggests that minor transcription errors in the fibroblast growth factor – 10 pathways, hedgehog signaling pathways, and Homeobox protein Nkx 2.1 may lead to localized anomalies of the bronchial cartilage resulting in CLE, since these are responsible for the branching morphogenesis of the lung [3].

The diagnosis of CLE is suspected with plain chest radiography. CT scans are useful for confirmatory purposes [10, 11]. Lobectomy is the treatment of choice, with excellent outcomes in most patients [12]. New techniques, such video-assisted thoracoscopy, promise a safer and faster surgical approach for this condition [12]. Bronchoscopy prior to any the surgical procedure tends to be very rewarding, as it permits the confirmation of bronchomalacia and continues with the planned surgical approach, or in some cases, find an extrinsic mass or endobronchial obstruction, and avoid lobectomy [13].

The use of endobronchial valves in patients with CLE and other forms of severe emphysema are controversial. Recent studies, including the VENT trial, have demonstrated that, in very selected patients, a complete single lobar obstruction using an IBV valve can be beneficial in improving the pulmonary function and clinical outcomes [14, 15]. Even though the role of the IBV valve or other endobronchial valves in bullous lung disease have not been thoroughly studied in well-randomized, controlled trials, this therapeutic intervention may offer a good nonsurgical alternative option in patients with CLE who cannot tolerate surgery [16].

## 3. Conclusions

CLE is a rare condition of unknown etiology rarely seen in adults. The diagnosis can be established with chest radiographs and high clinical suspicion. CT scans are used as confirmatory tests and allow for surgical planning. Lobectomy is the treatment of choice and has excellent outcomes. Newer techniques, such as VATS are emerging with good results. Endobronchial valves appear to be a potential option in the future.
